# Self‐Assembled Silicon@Silica Metasurfaces with High‐Quality Resonances in the Infrared

**DOI:** 10.1002/smsc.202500119

**Published:** 2025-05-15

**Authors:** Megan A. Parker, Raul Barbosa, Cynthia Cibaka‐Ndaya, Alexander Castro‐Grijalba, Maria Letizia De Marco, Brian A. Korgel, David Montero, Sabrina Lacomme, Antoine Azéma, Vasyl G. Kravets, Alexander N. Grigorenko, Virginie Ponsinet, Philippe Barois, Lucien Roach, Glenna L. Drisko

**Affiliations:** ^1^ University of Bordeaux, CNRS, Bordeaux‐INP, ICMCB, UMR 5026 33600 Pessac France; ^2^ McKetta Department of Chemical Engineering and Texas Materials Institute The University of Texas at Austin Austin TX 78712 USA; ^3^ Sorbonne Université, CNRS, Fédération de Chimie et Matériaux de Paris‐Centre, UAR 2482 75252 Paris France; ^4^ University of Bordeaux, CNRS, INSERM, Bordeaux Imaging Center, UAR 3420 33600 Pessac France; ^5^ LAAS‐CNRS Université de Toulouse 31000 Toulouse France; ^6^ CEMES‐CNRS Université de Toulouse 31000 Toulouse France; ^7^ Department of Physics and Astronomy University of Manchester Manchester M13 9PL UK; ^8^ University of Bordeaux, CNRS, CRPP, UMR 5031 33600 Pessac France

**Keywords:** 2D particle assembly, core‐shell particles, metasurface, Mie resonance, silicon, supercritical synthesis

## Abstract

2D assemblies of resonant dielectric particles constitute promising materials for the next generation of photonic devices, thanks to their low optical losses and intense electromagnetic response. However, bottom‐up synthesis methods present many difficulties when targeting metasurface applications, particularly due to the high degree of positional disorder and the size dispersion of the resonant particles. This work presents the fabrication of core–shell silicon@silica particles with multipolar resonances in the visible and near‐infrared. These resonant particles are then assembled at an air–water interface into a semi‐ordered array with islands of crystallinity. The assembly is deposited on quartz and the optical properties are characterized with ellipsometry and optical microscopy. The effective medium of this material appears to display a magnetic resonance with a high‐quality factor at ≈945 nm, as demonstrated by a Lorentzian resonance in the permeability. Thus, this is the first bottom‐up synthesis of silicon particle assemblies known to generate optical magnetism, giving promise for the scalable production of high‐performance metasurfaces, in spite of the imperfections associated with bottom‐up fabrication.

## Graphical abstract

An artificial magnetic resonance is created via the fabrication of a 2D silicon particle assembly. This particle to metasurface bottom‐up synthetic strategy presents many structural defaults and deviates largely from the ideal metasurfaces produced through top‐down techniques. Even so, the effective medium of this material appears to display a magnetic resonance with a high‐quality factor at ≈945 nm.

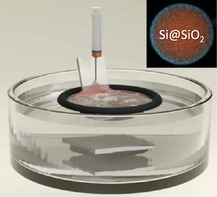

## 1 Introduction

Mie resonance within dielectric particles leads to strongly oscillating polarization currents under light irradiation, generating optical magnetism, which can cause intense light scattering with low losses.^[^ ^1^ ^]^ Metasurfaces composed of Mie resonant dielectric particle assemblies have been studied for applications in wave‐shaping,^[^ ^2^ ^]^ anti‐reflective surfaces,^[^ ^3^ ^]^ holograms,^[^ ^4^ ^]^ visual appearance,^[^ ^5^ ^]^ and miniaturized planar lenses.^[^ ^6^ ^]^ The rich diversity of applications is largely due to the sensitivity of the effective medium to particle–particle coupling. Coupling between resonant silicon particles has been studied thoroughly. As the scattering cross‐section is much larger than the particle diameter, coupling occurs even when particles are well separated.^[^ ^7^ ^]^ However, not all modes are equally impacted by particle spacing. For instance, the magnetic dipole is much less sensitive to particle–particle coupling than is the electric dipole,^[^ ^7^ ^]^ but the electric dipole of one particle can couple with the magnetic dipole of another. Such rich dynamics have been harnessed by García‐Cámara et al. creating nanometric switches by generating interferences between coupled dimers with two different diameters.^[^ ^8^ ^]^ Highly directional radiation patterns can be generated from Si nanodisk dimers, due to hotspots generated from the electric field and the third harmonic radiation from magnetic dipole coupling.^[^ ^9^ ^]^ Beyond the highly sensitive and diverse optical properties of Si dimers, larger particle arrays can be expected to present more opportunities to exploit their properties.

There are a few examples of arrays larger than Si dimers. Negoro et al. sequentially studied the optical scattering from monomers, dimers, trimers, and tetramers of spherical Si particles.^[^ ^10^ ^]^ As occurred in the previously reported dimers,^[^ ^7^, ^8^ ^]^ linear assemblies of four silicon particles illuminated with visible light demonstrate a broadened and redshifted electric dipole response, compared with a single particle. The magnetic dipole stays strongly confined within the particle with little coupling to neighboring particles. The forward/backward scattering ratio was improved by generating a small cluster of particles thanks to the better overlap between the electric and magnetic modes, upon broadening and red‐shifting the electric dipole. Top‐down techniques have confirmed that Si monolayers can be used as Huygens’ metasurfaces, but that positional disorder is an important factor in achieving enhanced forward/backward ratio by increasing the spread of induced dipole moments.^[^ ^11^ ^]^ Bulk multilayer metasurfaces have been produced using nonthermal plasmas to synthesize 80 nm diameter Si particles and then sprayed onto a substrate to create a loosely packed particle assembly.^[^ ^12^ ^]^ By calculating a modified effective medium, considering interparticle coupling and multipole resonances, the optical response of the films could be accurately described.^[^ ^12^ ^]^ Again, the optical magnetic resonance remained confined to the particle, where the electric dipole was much more heavily impacted by particle coupling. Monolayers of closely packed silicon particles have recently been realized for the first time, though the effective medium of the films was not characterized.^[^ ^13^ ^]^

While particle–particle coupling is a way to improve the forward/backward scattering ratio, this can also be achieved with an individual particle by creating a core–shell structure.^[^ ^14^ ^]^ We showed that broadband scatter in the visible could be achieved in single particles by creating a Si@SiO_*x*_N_*y*_ core–shell structure.^[^ ^15^ ^]^ In the present work, we have produced similar structures, but we have improved the crystallinity of the core, and therefore the strength of the resonances, by using cyclohexasilane instead of trisilane as one of the Si precursors used to create the particles. These particles were assembled into a monolayer at a liquid–air interface and then collected onto a substrate. We believe that we have observed for the first time a material fabricated via bottom‐up assembly with a very high‐quality factor magnetic resonance resulting from an effective medium, rather than individual particles.

## 2 Results and Discussion

**Figure 1:**
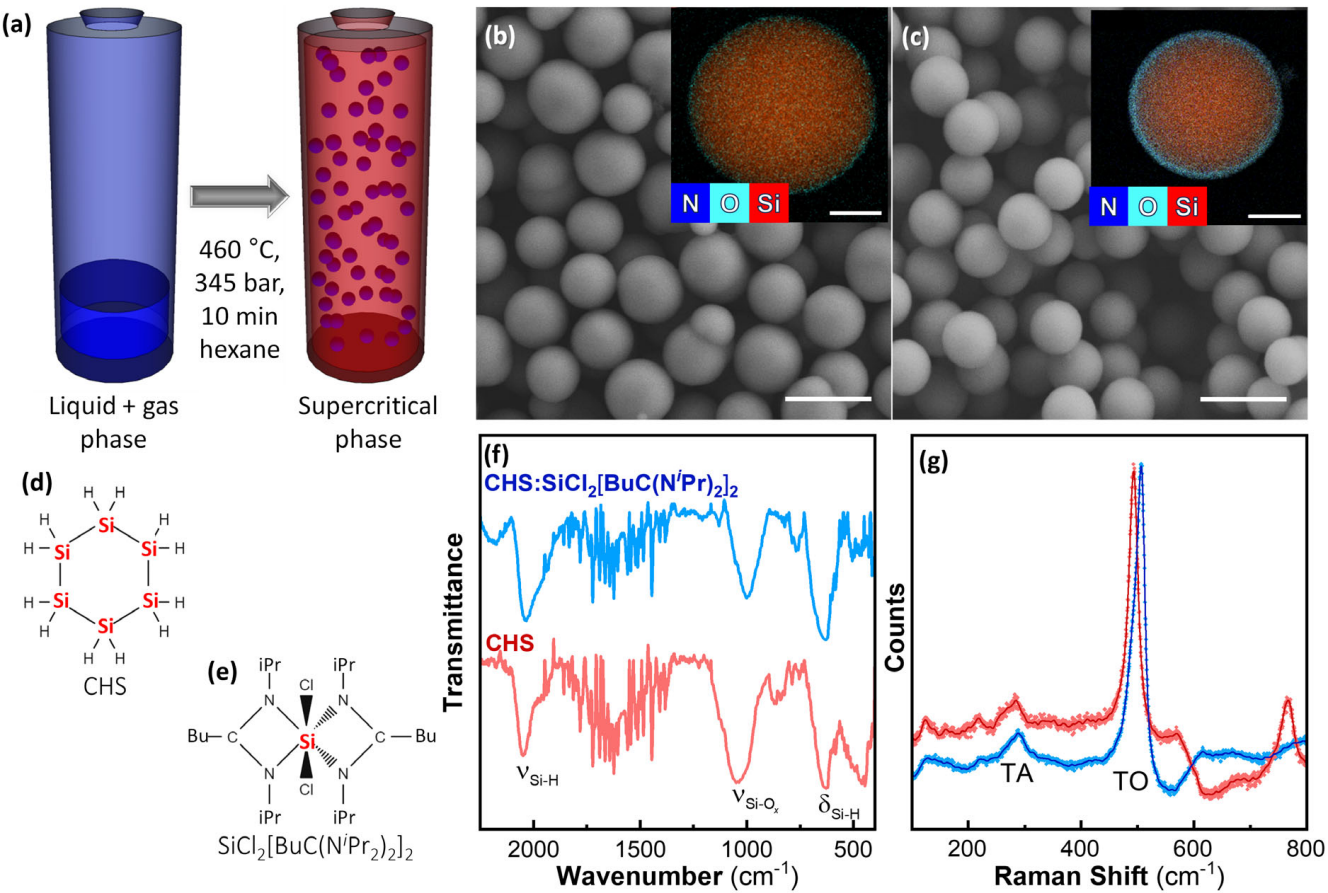
Synthetic strategy to produce resonant core–shell particles. a) Core–shell particles are produced in a batch synthesis in supercritical hexane heated to 460 °C in a titanium reactor. SEM micrographs of particles produced using b) CHS and c) a 3:1 molar ratio of CHS to SiCl_2_[BuC(N^*i*^Pr)_2_]_2_. The insets are of STEM‐EDX mapping of the particles with red representing Si, dark blue nitrogen, and light blue oxygen. The scale bars represent 1 µm for the SEM images and 200 nm for the STEM‐EDX images. Molecular structures of the two precursors used, that is, d) CHS and e) SiCl_2_[BuC(N^*i*^Pr)_2_]_2_. f) Fourier‐transform infrared transmittance spectra, where *δ* represents the bending modes and *ν* the stretching modes, and g) Raman spectra for particles synthesized using CHS (red) and a 3:1 molar ratio of CHS:SiCl_2_[BuC(N^*i*^Pr)_2_]_2_ (blue).

We produced Si particles by loading a titanium batch reactor at room temperature with two Si precursors, cyclohexasilane (CHS) and a Si coordination complex, bis(*N*,*N*′–diisopropylbutylamidinato)dichlorosilane (SiCl_2_[BuC(N^*i*^Pr)_2_]_2_), in *n*‐hexane under N_2_ (**Figure** **1**a). The reactor was placed in between heating blocks at 460 °C to quickly pass into the supercritical regime of hexane. At this temperature, the precursors rapidly decompose into both elemental and hydrogenated Si, leading to the reaction completing in ≈10 min. The particle synthesis was repeated 40 times to assess reproducibility in terms of particle size and dispersion. Although the polydispersity within a batch is typically low, the batch‐to‐batch variation is significant (Figure S1, Supporting Information). This is perhaps likely due to the challenge of handling small precursor volumes. Unlike trisilane, CHS has lower volatility, which may improve batch‐to‐batch reproducibility. We studied two systems, pure CHS and CHS combined with SiCl_2_[BuC(N^*i*^Pr)_2_]_2_, both produced spherical Si particles (Figure 1b,c). There are several noticeable differences between these systems. The average diameter of the CHS particles (775 nm) is larger than the CHS + SiCl_2_[BuC(N^*i*^Pr)_2_]_2_ particles (540 nm). The size polydispersity is also higher in the pure system, with a standard deviation in the size dispersion of 16%, as opposed to 9% for the CHS + SiCl_2_[BuC(N^*i*^Pr)_2_]_2_ batch. Moreover, particle necking and aggregation are mostly avoided when cyclohexasilane is combined with the Si coordination complex.

Another difference between the particles generated by the precursor pair and the CHS precursor is the thickness of the oxidation layer around the Si core. The thickness of the shell for both systems was observed by scanning electron microscopy (SEM)/scanning transmission electron microscopy‐electron dispersive X‐ray (STEM‐EDX) mapping (Figure 1b,c). In the case of the pure CHS particles, this layer is only a few nm in thickness, which results from passive oxidation. In the co‐precursor synthesis, there is a more prominent shell of ≈25 nm in thickness. We have previously hypothesized that as the temperature rapidly increases, the Si precursors start to decompose to form elemental Si, followed by the decomposition of the amidinate ligand at temperatures above 250 °C.^[^ ^15^ ^]^ Fragments of this ligand may interact with the non‐reacted Si precursor, leading to the formation of Si—N bonds and a less dense Si structure. Upon cooling and exposing the sample to air, the more porous outer sections of the Si particles oxidize to form an oxide shell. However, this shell is thinner for the particles produced from CHS + SiCl_2_[BuC(N^*i*^Pr)_2_]_2_ than it was for the particles that we previously produced from trisilane + SiCl_2_[BuC(N^*i*^Pr)_2_]_2_, perhaps because CHS is reacts at a lower temperature, or because it is much more reactive than trisilane.^[^ ^15^ ^]^ Infrared spectroscopy showed a difference in the intensity of the Si‐O_*x*_ vibration mode (≈1050 cm^−1^) between the particles formed from CHS and those formed from CHS + SiCl_2_[BuC(N*^i^* Pr)_2_]_2_, as observed with the presence of an oxide shell in STEM‐EDX mapping (Figure 1f). Si‐H_*x*_ vibrational modes (≈660 and 2100 cm^−1^) indicate the presence of hydrogen in both particle types. The oxide shell is beneficial, allowing the particles to be easily surface‐functionalized and dispersed in alcoholic solution.

The final difference between the particles produced from the pure and the co‐precursor systems is the degree of bond order. Two Si–Si vibrational modes can be observed in the Raman spectra of the samples: the transverse optical (TO) mode between 480 and 520 cm^−1^, and the transverse acoustical (TA) mode around 300 cm^−1^ (Figure 1g). Raman spectroscopy is highly sensitive to short‐range order and thus is commonly used to assess Si systems that appear amorphous by X‐ray diffraction. A sharp peak at 520 cm^−1^ indicates crystalline Si, whereas a broader band at 490 cm^−1^ indicates amorphous Si.^[^ ^16^ ^]^ In Figure 1g, we see that the CHS particles display a peak at 493 cm^−1^, and the particles from the co‐precursor system display a peak at 507 cm^−1^. Particles produced from combining CHS + SiCl_2_[BuC(N^*i*^Pr)_2_]_2_ demonstrate a higher degree of bond order, meaning a more crystalline character, than the CHS particles.

The particle size resulting from both the pure CHS and the co‐precursor synthesis depended on the precursor concentration used. The Si particle diameter in the CHS system varied between ≈700 nm and 1 μm by varying the CHS concentration in the supercritical reactor in the range of 5–15 μm. In the co‐precursor system, for a 1:3 CHS: SiCl_2_[BuC(N^*i*^Pr)_2_]_2_ ratio, and at the same concentration as for pure CHS, the particle diameter was ≈200 nm smaller. However, we did not synthesize enough batches as a function of concentration to be able to report precise values for the particle diameter. We believe that the particles are smaller when CHS and SiCl_2_[BuC(N^*i*^Pr)_2_]_2_ are co‐decomposed because nucleation is more favored due to the high reactivity between the silylene intermediates produced from CHS ring‐opening,^[^ ^17^ ^]^ and the Lewis acid center of the coordination complex. The ratio between precursors was fixed to 1:3 CHS:SiCl_2_[BuC(N^*i*^Pr)_2_]_2_ with the concentration of CHS held constantly at 5.5 μm. The synthesis was repeated several times to produce enough particles to fabricate a continuous monolayer.

**Figure 2:**
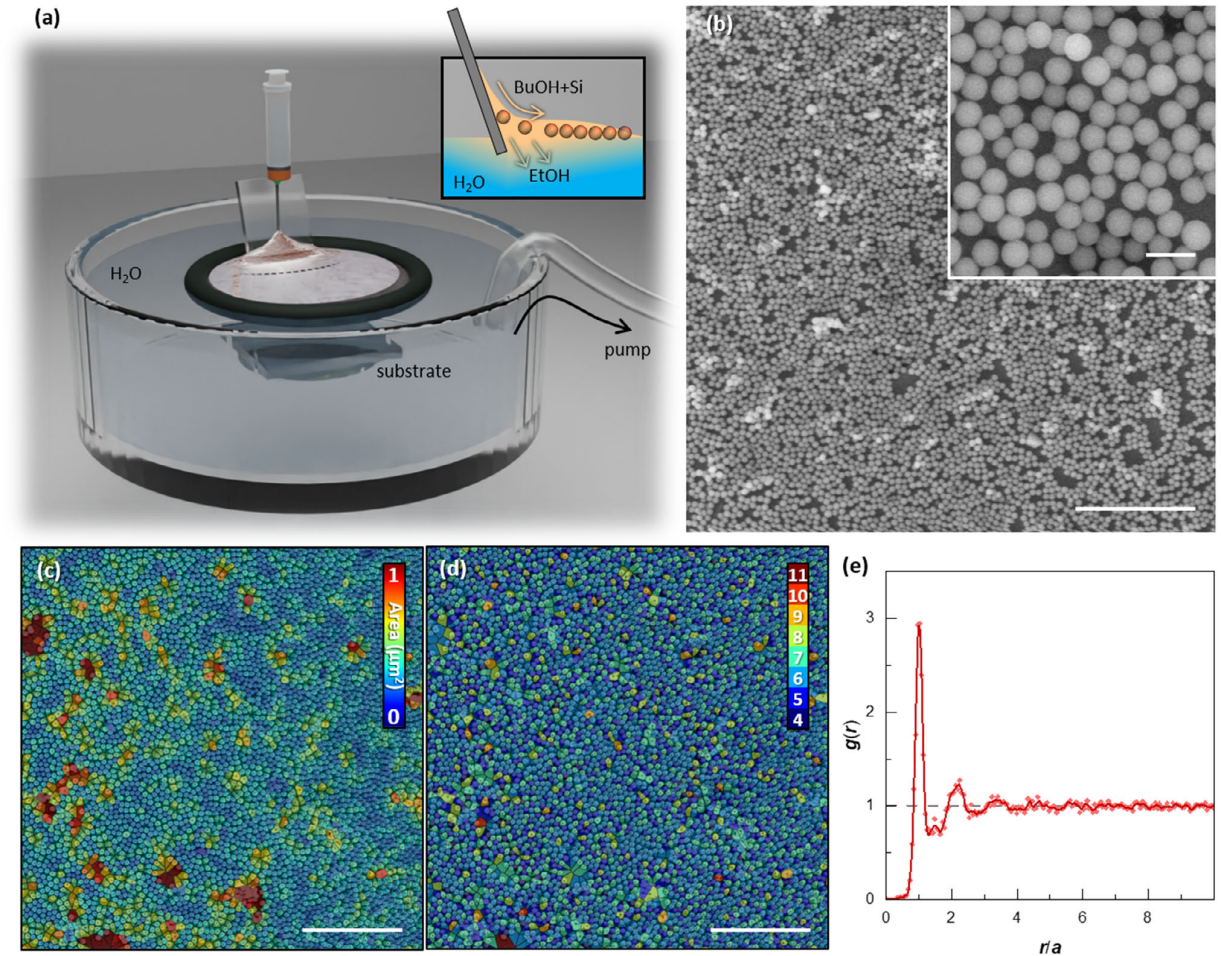
Assembly of core–shell particles into a monolayer. a) Experimental set‐up with (inset) schematic of the interfacial assembly process with particles dispersed in 2:1 ethanol:butanol, lodging themselves at the surface of the aqueous bath. b) SEM images of a surface covered with the particles, where the monolayer is nearly continuous and a small fraction of the film is multilayer. The scale bar represents 10 μm and the inset scale bar represents 1 μm. Analysis of packing density: c) areas of the Voronoi cells, d) the number of nearest neighbors, and e) the correlation function.

To fabricate a large area metasurface (≈1 cm^2^) composed of resonant Si@SiO_2_ core@shell particles, an interfacial self‐assembly technique was developed to deposit a monolayer of resonators on a quartz substrate. The supercritical synthesis of these particles in *n*‐hexane results in slightly hydrophobic surface chemistry. To re‐disperse the particles in alcohols, the particles were functionalized with an amphiphilic block copolymer (Pluronic F‐127). To trap the particles at the surface of a water bath, the particles were first suspended in a solution of 2:1 ethanol:butanol and then ejected through a syringe onto a hydrophilic glass microscope slide inserted at a slightly obtuse angle into the surface of a water bath (**Figure** **2**a). The high miscibility of ethanol in water leads to immediate ethanol solvation into the aqueous phase, whilst the butanol–particle mixture is trapped at the interface and distributes over the H_2_O surface. The thin particle layer is confined to a region enclosed by a nitrile rubber ring. The particles can then be transferred to a hydrophilic substrate by draining the water bath using a pump. The prepared films were fairly closely packed and uniformly disordered (Figure 2b).

The disorder within a film can be quantified in different ways, including nearest neighbor distances, number of nearest neighbors, and correlation function (Figure 2c,d). In order to quantify the disorder in these samples, an SEM image was segmented to binarize the particles and the substrate, then the particle coordinates were extracted (Figure S2, Supporting Information). The particle distribution was then characterized through Voronoi–Delaunay analysis. The area of individual Voronoi cells was calculated, and a color scale was applied to the area of each cell. For a hexagonally close‐packed lattice of 540 nm diameter particles, an ideal Voronoi cell would have an area of 0.25 μm^2^. Cells with this area appear in light blue in Figure 2c, whereas the median Voronoi area is 0.29 μm^2^ (Figure S3a, Supporting Information). Smaller than ideal cell areas can be achieved by smaller particles. In Figure 2d, the number of closest neighbors is indicated, where a perfectly close‐packed crystal should have six neighbors for each particle (light blue cells). The median number of nearest neighbors is 5.9 (Figure S3b, Supporting Information). As can be seen in Figure 2b, voids are largely responsible for a deficit of nearest neighbors, whereas aggregates are responsible for particles with high numbers of nearest neighbors, because they are partially organized in three dimensions. There is a peak in the distribution of nearest neighbor distances equivalent to one particle diameter (540 nm) (Figure S3c, Supporting Information), with a smaller peak in nearest neighbor distances having ≈700 nm. This distance corresponds to the diagonal distance in a square packed lattice. The correlation function was calculated to indicate the statistical distribution of the particles at any point. We can observe three, perhaps four, peaks in the radial distribution, indicating short‐range order over multiple particles. Later we will explore whether this particle density and degree of correlation is high enough to generate ensemble properties.

**Figure 3:**
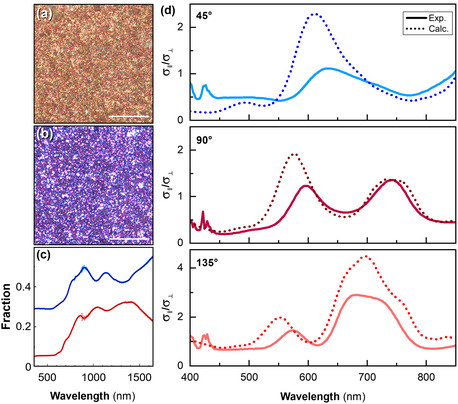
a) Reflection and b) transmission optical microscopy images of the self‐assembled particle films prepared in Figure 2. The scale bars represent 4 μm. c) UV‐vis‐NIR transmission (blue) and reflection (red) spectra of the same sample. d) Axial to transverse ratio of the light scattered by dilute suspensions of core–shell particles collected at scattering angles of *θ* = 45°, 90°, and 135°, with the experimental ratio, *σ*_∥_(*λ*)/*σ*_⊥_(*λ*), (solid lines) and calculated spectra (dotted lines).

Optical microscopy images of the films were taken in transmission and reflectance mode using a white light source for illumination (**Figure** **3**a,b). The recovered images of the particle film were predominantly blue in transmission and predominantly red in reflection. This is supported by ultraviolet‐visible‐near infrared (UV‐vis‐NIR) spectroscopy of the film, which showed that the film more strongly transmitted blue light and more strongly reflected red light (Figure 3c), indicating that these films were acting as filters.

To understand the optical scattering modes of these films in the visible, we performed polarization‐resolved static light scattering (SLS) on dilute suspensions of particles in chloroform using a custom‐built set‐up (Figure S4, Supporting Information). This technique measures the intensity of scattered light at a known angle relative to incident light and for two polarization states, namely perpendicular to the scattering plane (referred to as transverse or *I*_sca,⊥_) and parallel to the scattering plane (parallel or *I*_sca,∥_). The experimental measurements were analyzed using Mie theory which describes the scattering by a coated sphere in terms of a multipolar expansion.^[^ ^18^ ^]^ Polarization‐resolved measurements enable the identification of the scattering modes. When the scattering angle is set to 90°, odd and even modes of the Mie expansion are decoupled,^[^ ^19^ ^]^ which helps to identify the successive resonances in the spectral landscape.^[^ ^20^ ^]^ However, since the resonant particles were relatively large compared to the visible wavelengths, particularly considering their high refractive index, the dipole resonances occur in the NIR, outside of the detection window of this apparatus. Nevertheless, peaks derived from resonances of higher multipoles occur within the visible spectrum. Three collection angles (*θ* *= *45°, 90°, and 135°) were used to help strengthen the comparison between measurements and calculations, and the comparison was considered, for each scattering angle, on the ratio *σ*_‖_/*σ*_⊥_ of the parallel (∥) and transverse (⊥) differential scattering cross‐sections (Figure 3c). In all cases, insignificant light scattering occurred below 500 nm, thus explaining the blue light transmission observed by optical microscopy (Figure 3b). However, there were scattering peaks at longer wavelengths at all collection angles observed, consistent with the observed reflectance in the red part of the spectrum (Figure 3a and S4, Supporting Information).

Axial and transverse scattering were calculated using Mie theory. Several known parameters were used to refine the adjustment with measured data: a measured shell thickness of 25 nm was applied, as well as the size distribution histogram measured from SEM images. The highly crystalline nature of the core indicated by Raman spectroscopy was considered. With this information, a model of the refractive indices of the core and the shell was created with the Maxwell‐Garnett mixing rule. Specifically, for both core and shell, we mixed the refractive index of crystalline Si with amorphous Si and with a low‐index material meant to account for porosity or silica inclusions (Table S1, Supporting Information). By testing different proportions of these three components for each of the two indices, a relatively good fit for all three collection angles was obtained (Figure 3d). The good agreement between simulations and experiments provides a strong argument for a dispersion composed of well‐dispersed, non‐aggregated particles.

The resulting refractive index for the particle core material is shown in Figure S5, Supporting Information. In keeping with the high bond order in these particles, the refractive index is estimated to be around 4 across the visible, with a low‐absorption coefficient (*κ*). The refractive index is slightly lower than pure‐crystalline Si and slightly more absorbing, but has dramatically better optical scattering properties than amorphous Si and the particles that we previously reported prepared from trisilane and SiCl_2_[BuC(N^*i*^Pr)_2_]_2_.^[^ ^15^ ^]^ The refractive index of the shell is close to silica (*n*_shell_ ≈ 1.41) at 500 nm and has a very low‐absorption coefficient (Figure S5, Supporting Information). Thus, it is clear that these materials demonstrate multiple intense magnetic and electric resonances with low absorption, therefore they make good building blocks for the construction of a metasurface. The next step is then to try to measure the refractive index, and therefore magnetic permeability and electric permittivity of the 2D assembly.

**Figure 4:**
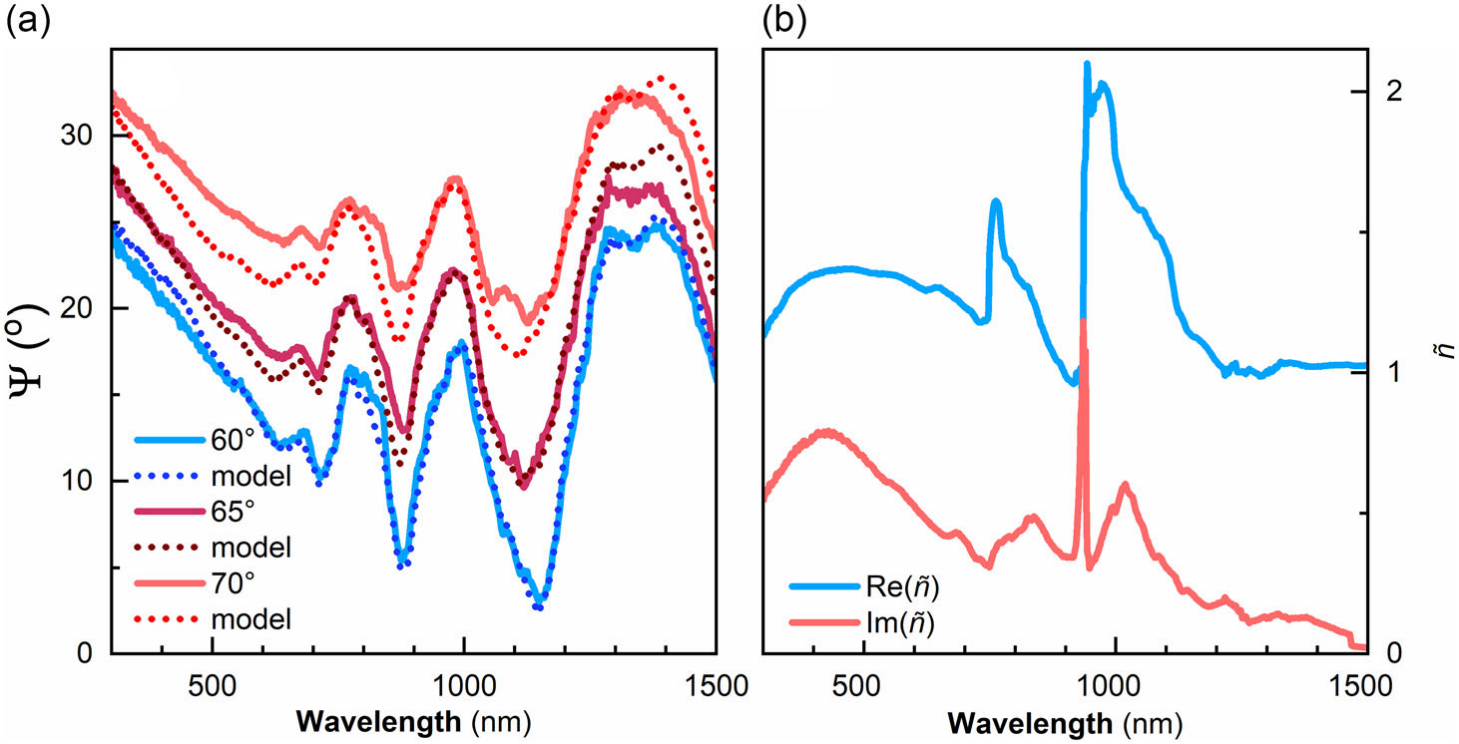
a) Fresnel fitting of the variable angle ellipsometry spectra using the *Meta6* model with 540 nm diameter particles and a quartz Cauchy substrate of 1 mm at 60°, 65°, and 70°. Experimental data plotted (solid lines) versus modeled spectra (dotted lines). Data for 50° and 55° can be found in the supporting information (Figure S6, Supporting Information). b) Real and imaginary parts of the refractive index (Re(*ñ*) = *n* and Im(*ñ*) = *κ*) of the Si@SiO_2_ core–shell particle monolayer.

Variable angle spectroscopic ellipsometry was used to characterize the assembled monolayer of Si particles. Variable angle spectroscopic ellipsometry allows one not only to measure the far‐field reflection of the samples (including Muller matrices) but also to check whether the studied metamaterials could be described by effective constants (permittivity and permeability). We measured the ellipsometric parameters Ψ and Δ defined as *r*_∥_/*r*_⊥_ = tan(Ψ) exp(*i*Δ), where *r*_∥_ = *E*_r,∥_/*E*_i,∥_ and *r*_⊥_ = *E*_r,⊥_/*E*_i,⊥_ are the reflection coefficients for light polarization parallel, and perpendicular to the plane of incidence, *E*_r_ and *E*_i_ denote the electrical field of the reflected and incident light, respectively.^[^ ^21^ ^]^ By definition, it follows that Ψ goes to zero whenever p‐polarized reflection goes to zero and Ψ is equal to 90° whenever *s*‐polarized reflection is zero. Despite the inhomogeneities within the fabricated samples, the low scattering of the films made fitting possible. We obtained a good fit using the *Meta6* layer model for optical spectra measured at five different incidence angles (**Figure** **4**a and S6, Supporting Information), a model that has been previously used to confirm non‐trivial permeability in plasmonic dimers.^[^ ^22^ ^]^ The fit had a reasonable mean square error (*MSE* = 26), suggesting that the monolayers of large dielectric particles can be described by local constants. The fit process is done by adjusting the values of *ε* and *μ*, where possible crosstalk effects in the fit of the ellipsometry data can be reliably circumvented through their combination in the refractive index $n=\sqrt{\varepsilon \mu }$ (Figure 4b).

**Figure 5:**
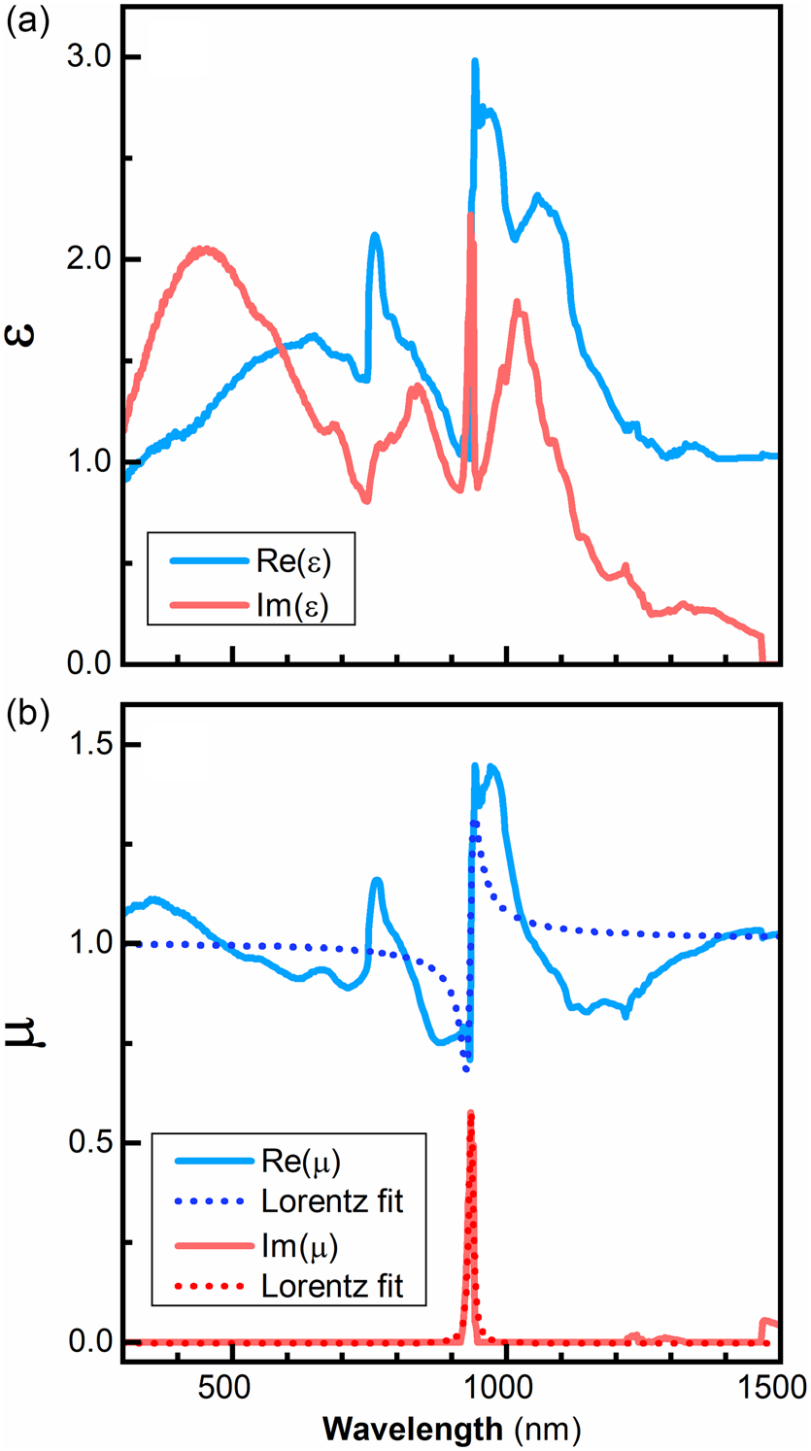
Complex a) permittivity and b) permeability of a core–shell Si@SiO_2_ core–shell particle monolayer. The real parts are in blue and the imaginary parts are in red.

Figure 4b shows the extracted values of *n* and *κ* for the studied monolayer of dielectric particles. Magnetic and electric resonances are observed in the 800–1200 nm range. The main feature shows a sharp resonance at ≈945 nm. In **Figure** **5**, we present the complex permittivity (*ε*) and permeability (*μ*) that we extracted for the studied Si@SiO_2_ core–shell particle monolayers, which correspond to the refractive index presented in Figure 4. Both *ε* and *μ* were found to be isotropic, while chirality was found to vanish at all wavelengths. There is a strong crosstalk between permeability and permittivity extracted values at ≈945 nm. Using an Accurion software, we checked that the crosstalk was below 50%, which allows us to separate the individual contributions from permittivity and permeability. After this separation, we can see that there is a strong, Lorentz‐shaped resonance in the permeability (Figure 5b), suggesting the presence of the magnetic resonance at 945 nm. This magnetic response differs from natural materials, where *μ* = 1 for all wavelengths. To double check, we performed fitting of the ellipsometry spectra of the monolayer sample with a constraint of *μ* = 1, which did not provide a good fit to the measured data. The underlying mechanism enabling optical magnetism in fabricated metasurfaces is a magnetic Mie resonance in silicon particles. It is exactly the same mechanism as for the optical magnetism observed in clusters of silicon particles.^[^ ^12^ ^]^ The presence of large areas with highly crystalline order within the fabricated metasurfaces ensured high‐quality resonances and made it possible to describe the metasurface as an effective medium.

To quantify the intensity of this resonance, we use a quality factor defined as *Q* = *λ*_MR_/Δ*λ*_MR_, where Δ*λ*_MR_ is the full‐width‐at‐half‐maximum of the resonance. The quality factor of the magnetic resonance at ≈945 nm yields a value of *Q* ≈ 62. Since here we are dealing with photon energies above the bandgap of silicon, where silicon is not dielectric, this resonance quality is very high. In comparison, the magnetic resonance in the silicon particles observed in Ref. 12 at wavelengths of ≈450 nm (above bandgap energy) had a quality of just *Q* ≈ 10.

The importance of a large value of *Q* can be understood from the following considerations. Suppose we are looking for a low‐absorption metamaterial. If we fix the value of the imaginary part of magnetic permeability as Im(*μ*) < *r*, where *r* is a small number, then the maximal value of the real part of the magnetic permeability achieved by Mie‐resonance will be around |Re(*μ* − 1)| ≈ $\sqrt{Q/f}$, where *f* is the oscillator strength. A figure of merit (*FOM*) for this material can be defined as *FOM* = |Re(*μ* − 1)/Im(*μ*)| ≈ $\sqrt{Q/rf}$. Hence, the higher the value of the quality *Q*, the larger the value of the permeability we achieve under fixed losses. One possible hypothesis is that the high *Q* can be attributed to small islands of ordered particles of similar size. Ideally, simulations would confirm this extraordinary result, but it is difficult to model ellipsometry conditions with large particles in a large, non‐periodic array on a substrate. Future work will thus focus on semi‐infinite simulations of reflection and transmission.

## 3 Conclusion

Dielectric particle metamaterials could solve the problem of losses inherent to plasmonic particles, providing metamaterials with high‐quality magnetic resonances. Thus, we have prepared batches of resonant Si@SiO_2_ core–shell particles to be used as building blocks. By simultaneously decomposing cyclohexasilane and SiCl_2_[BuC(N^*i*^Pr)_2_]_2_, we have achieved Si cores with a large degree of crystalline character. Combining these two precursors decreases the average size, decreases the size dispersion within a batch, and increases the crystalline character as compared to cyclohexasilane on its own. Previous studies decomposing trisilane and SiCl_2_[BuC(N^*i*^Pr)_2_]_2_ produced more amorphous silicon particles, with smaller average diameters and a thicker oxide shell. Here, 540 nm diameter core–shell particles were produced with a 25 nm oxide shell.

Multimodal resonances of the core–shell particles were optically characterized using polarization‐resolved static light scattering on a particle suspension in chloroform. We collected light scattered at three different angles (45°, 90°, and 135°) to ensure the quality of the fit. We then extracted the refractive index and absorption coefficient, showing a refractive index close to bulk Si and low absorption. Strong resonances are found to occur in the red part of the visible spectrum, which can thus explain why these films reflect red and transmit blue light.

Monolayer 2D assemblies of the particles were created through interfacial air–liquid assembly and then collected on a quartz substrate of ≈1 cm^2^. These deposited films were partially ordered with high packing density. A majority of particles had 6 nearest neighbors, with an average bond length equivalent to the particle diameter. The optical properties of this film were measured with variable angle spectroscopic ellipsometry and fitted as a homogenized layer with an effective medium to extract permittivity and permeability. The main feature identified was a sharp and strong resonance in permeability, with a high quality factor, meaning that absorption was relatively weak compared to the strength of the signal. This metasurface is therefore the first produced through bottom‐up synthesis using Si building blocks that seems to show *μ* ≠ 1. This is intriguing because, normally, structural resonances require a high degree of structural correlation, and we have a certain degree of dispersity in position, size, and shape. Unfortunately, we cannot describe the results with a model at this time, because of the difficulty simulating this non‐perfect system. However, the results indicate that plasmonics can be feasibly replaced in metasurfaces by Si particles. Future work will focus on improving the monodispersity of the particles and increasing the structural correlation between them.

## 4 Experimental Section

4.1

4.1.1

##### Materials

*N*,*N*′–diisopropylcarbodiimide (99%), Si tetrachloride (99%), *n*‐butyl‐lithium (2.5 m in hexane), *n*‐hexane (anhydrous 95%), chloroform, ethanol, butanol, Pluronic F–127, and isopropyl alcohol were purchased from Sigma‐Aldrich and used without further purification. Toluene and THF were dried using an Innovative Technology solvents purification system, then oxygen was removed by three freeze‐pump‐thaw cycles. The purified solvents were stored over 3 Å molecular sieves in an Ar‐filled glovebox. Cyclohexasilane was obtained from North Dakota State University, and stored in a glovebox (O_2_ level < 0.1 ppm).^[^ ^23^ ^]^

##### Synthesis of Bis(N,N′–Diisopropylbutyl)Dichlorosilane

The synthesis of bis(*N*,*N*′–diisopropylbutyl)dichlorosilane, [(*i*Pr–N)_2_C(C_4_H_9_)]_2_SiCl_2_ was carried out under an inert atmosphere, using Schlenk techniques to protect reactants and products from moisture and oxygen, according to previously published protocols.^[^ ^24^ ^]^ Butyl‐lithium (13 mL, 0.032 mol) was added to an *N*,*N*′‐diisopropylcarbodiimide solution (5 mL, 0.032 mol) in THF (80 mL) at −30 °C under magnetic agitation. This reaction yields lithium (*N*,*N*′–diisopropylbutylamidinate), [(^*i*^PrN)_2_C(CH_3_)]Li·THF. The reaction was stirred overnight at room temperature to ensure a complete reaction. Then, SiCl_4_ (1.86 mL, 0.016 mol) was added at −78 °C. The solution was stirred at room temperature for 18 h. The solvent was evaporated and substituted with toluene (60 mL). The solution was filtered, and bis(*N,N*′–diisopropylbutyl)dichlorosilane was crystallized from the filtrate by slow evaporation of the toluene, and then was stored at −15 °C, under an argon atmosphere, to avoid premature degradation, as it is extremely sensitive to moisture and thermally labile.

^1^H NMR (400 MHz, C_6_D_6_, 25 °C): Δ 0.77 (t, 6H *n*‐Bu, ^3^J_H‐H_ 7.44 Hz, CH_3_), *δ* 1.1 (d, 6H *i*‐Pr, ^3^J_H–H_ 6.52 Hz, CH_3_), *δ* 1.13 (m, 4H *n*‐Bu, ^3^J_H–H_ 6.8 Hz, MeCH_2_), *δ* 1.39 (d, 6H, ^3^J_H–H_ 6.68 Hz, CH_3_), *δ* 1.38 (m, 4H, *n*‐Bu, MeCH_2_CH_2_), *δ* 1.60 (d, 6H, ^3^J_H–H_ 7.00 Hz, CH_3_), *δ* 1.63 (d, 6H *i*‐Pr, ^3^J_H–H_ 6.88 Hz, CH_3_), *δ* 2.01 (m, 4H *n*‐Bu, CH_2_CN_2_), *δ* 3.50 (sept., 2H *i*‐Pr, ^3^J_H–H_ 6.76 Hz, Me_2_CH), *δ* 4.18 (sept., 2H *i*‐Pr, ^3^J_H–H_ 8.92 Hz, Me_2_CH).

##### Supercritical Synthesis of the Si@SiO_*2*_ *Core‐Shell Particles*

The supercritical synthesis was carried out in a 10 mL titanium (alloy grade 2) batch reactor, purchased from high‐pressure equipment (HiP, part number 210617). This reactor can withstand pressures and temperatures up to 345 bar (5000 psi) and 600 °C. The synthesis protocol was modified from our previous publication.^[^ ^15^ ^]^ The reactor was loaded in a N_2_ filled glovebox ([O_2_] <0.1 ppm) with *n*‐hexane (5.6 mL), cyclohexasilane (5.5 μL, 30 μmol), and bis(*N,N′*–diisopropylbutyl)dichlorosilane (28 μL, 10 μmol), (Caution! Cyclohexasilane is pyrophoric and must be handled with extreme care under inert atmosphere). The reactor was sealed tightly inside the glovebox, before being removed. The reactor was placed in a ceramic heating block that was preheated to 490 °C, using a dummy reactor. Supercritical synthesis was carried out at 460 °C and 300 bar, for 10 min. The reactor was then removed from the heating block and placed in an ice bath to quench the reaction. Once the reactor cooled to room temperature, it was opened and the products were collected under air. The resulting product was centrifuged at 8771 × g for 5 min, to precipitate the particles and discard the solvent. This cycle was repeated two more times, using chloroform to wash the products. The supercritical reaction yields about 6–9 mg of dry particles, which corresponds to a mass yield of about 20%.

##### Self‐Assembly of Si@SiO_*2*_ *Core–Shell Particles into a Monolayer*

A monolayer of the core–shell particles was prepared at an air–water interface before being deposited onto a quartz substrate by modifying a previously published protocol.^[^ ^25^ ^]^ The particles were mixed (1:1) with a 1 g L^−1^ Pluronic F‐127 solution, before washing by centrifugation and resuspension in a 2:1 ethanol:butanol mixture at a particle concentration of 2 % v/v. The particle suspension was introduced to a reservoir of water using a syringe pump. The solution was delivered to the surface of the water via a needle attached to a hydrophilic glass slide submerged slightly below the water surface at a slightly obtuse angle. The high miscibility of ethanol in water leads to this component immediately mixing with the aqueous phase, whilst the butanol–particle mixture becomes trapped at the surface and distributes over the water surface in a thin layer. A hydrophobic nitrile rubber O‐ring (of 4 cm diameter) suspended on the liquid surface was used to confine the forming particle film above a submerged quartz substrate. The addition of the particle suspension was stopped upon reaching a predetermined volume, equivalent to the number of particles needed to form a hexagonally close‐packed monolayer within the O‐ring. The water was then slowly removed by a peristaltic pump and the particle film was lowered onto the substrate before being allowed to dry. The films were plasma‐cleaned with a Harrick plasma cleaner (Model PDC 002 HPCE) until any remaining solvent residues were no longer visible under optical microscopy.

##### Nuclear Magnetic Resonance (NMR) Spectroscopy

Proton nuclear magnetic resonance spectra (^1^H NMR) of the Si bisamidinate were acquired using a Bruker Advance III‐HD 400 MHz SB spectrometer (Wissembourg, France) equipped with a 5 mm broadband SmartProbe at 25 °C in C_6_D_6_. ^1^H NMR spectra were acquired at 400.13 MHz using a single pulse sequence (*π*/2 pulse width of 10 μs, recycling delay of 2 s, acquisition time of 1.6 s, spectral window of 25 ppm, and 24 scans).

##### Fourier‐Transform Infrared (FTIR) Spectroscopy

FTIR spectra of the particles were collected using a Bruker Vertex 70 FTIR Spectrometer. A colloidal solution of particles in chloroform was drop‐cast onto a double‐polished Si wafer support. The sample was then placed in the optical path of the beam. During the measurement, a stream of nitrogen was flushed through the measurement chamber to evacuate CO_2_ and water vapor. The infrared spectra have been normalized with respect to the Si‐H_*x*_ stretching mode (≈2100 cm^−1^).

##### Scanning Transmission Electron Microscopy and Energy Dispersive X‐Ray Spectroscopy (STEM‐EDX)

STEM‐EDX observations were performed using a Thermofisher Talos F200S G2 FEG TEM equipped with a field emission gun operated at 200 kV, and a Gatan OneView camera (16 MP). The different TEM modes used are: Brightfield and high resolution up to 650 000 × magnification to image the morphology and dimensions of the particles. Their chemical composition was analyzed using energy‐dispersive X‐ray spectroscopy (EDS) in HAADF‐STEM. For the HAADF imaging, the annular detector was set to collect the electrons scattered at angles between 29–177 mrad (camera length, CL = 260 mm). For STEM/X‐EDS mode, the operational parameters are a condenser aperture, C2, of 50 μm (converge angle of 7.5 mrad), a current ≈200 nA to optimize the spatial resolution and X‐ray photon rate (around 1000 s^−1^). The TEM pictures were processed using DigitalMicrograph software (Gatan). STEM pictures and X‐EDS spectra and maps were processed using Velox software (Thermofisher). The TEM/STEM images and the spectra/elemental maps have been converted into TIF format after their post‐processing in the software used.

##### Raman Spectroscopy

Raman spectra were collected with an Xplora spectrometer by Horiba, equipped with a confocal microscope. An Olympus LM Plan FLN 100× objective lens with 0.80 numerical aperture and 3.4 mm working distance was employed to focus the laser beam on the sample and collect the scattered light. The spectra were captured using a 785 nm excitation wavelength, and a 25% neutral density filter. Final spectra were averaged from 15 spectra acquired using an acquisition time of 15 s. A 600 mm^−1^ diffraction grating was used to separate the scattered light into its components that were collected onto a Syncerity TE‐cooled FI‐UV‐VIS detector. Each spectrum was normalized by dividing by the area under each curve. Three measurements in three different zones were collected for each sample, to assess the reproducibility of the measurement and the uniformity of the sample.

##### Scanning Electron Microscopy (SEM)

SEM images were obtained using a Hitachi SU‐70. Samples were sputter coated with a thin gold layer (less than 10 nm) before image acquisition. Images were acquired with a 15 kV acceleration voltage. The quantification of the particle diameters from SEM images was performed using the software ImageJ. Analysis of the films was performed by first segmenting the images using ImageJ, then extracting the XY coordinates of the particles using ImageJ's “Analyze Particles” function. The extracted particle coordinates were then analyzed using a custom Python script. Voronoi and Delaunay analysis were performed using the SciPy.spatial module.

##### Ultraviolet/Visible/Near Infrared (UV‐Vis‐NIR) Extinction Spectroscopy

Reflectance and transmittance spectra were recorded using a Shimadzu UV‐3600 UV‐vis‐NIR spectrometer equipped with an integrating sphere attachment (ISR‐3100).

##### Polarization‐Resolved Static Light Scattering (SLS)

SLS was carried out using a custom‐built set‐up. The experimental geometry is shown in Figure S4, Supporting Information. We use the scattering matrix formalism,^[^ ^18^ ^]^ which links the scattered field *E*_s_ at a distance *r* of a scatterer to the incident field *E*_i_ (Equation (1)).

(1) $$\left(\begin{array}{c} E_{\text{s,}∥}\\ E_{\text{s,}⊥} \end{array}\right)=\frac{e^{ikr}}{-ikr}\left(\begin{array}{cc} S_{2}\left(\theta \right) & 0\\ 0 & S_{1}\left(\theta \right) \end{array}\right)\left(\begin{array}{c} E_{i}\text{cos}\varphi \\ E_{i}\text{sin}\varphi \end{array}\right)$$

where *k* is the wavevector. The plane defined by the directions of the incident and scattered beams represents the scattering plane. The subscripts ∥ and ⊥ stand for the field components with polarization parallel (axial) and perpendicular (transverse) to the scattering plane, respectively. For isotropic scatterers, such as spheres, the off‐diagonal elements of the scattering matrix are zero. Theta (*θ*) is the scattering angle and phi (*φ*) is the angle between the scattering plane and the direction of the polarization of incident light. At *θ* = 90°, the odd and even scattering modes separate: the *S*_1,⊥_ component cumulates the scattering contribution of the electric dipole, magnetic quadrupole, electric octupole, etc. while the *S*_2,∥_ component contains the contribution of the magnetic dipole, electric quadrupole, magnetic octupole, …etc.^[^ ^18^, ^19^, ^20^, ^26^ ^]^

We measured the intensities, $\tilde{I_{∥}}\left(\lambda ,\theta \right)$ and $\tilde{I_{⊥}}\left(\lambda ,\theta \right)$ (see Equation (3) and (4)) and extract the ratio

(2) $$\frac{\tilde{I_{⊥}}\left(\lambda ,\theta \right)}{\tilde{I_{∥}}\left(\lambda ,\theta \right)}=\frac{\sigma_{⊥}\left(\lambda ,\theta \right)}{\sigma_{∥}\left(\lambda ,\theta \right)}$$

where $\sigma_{⊥}\left(\lambda ,\theta \right)=\frac{{\left|S_{1}\left(\lambda ,\theta \right)\right|}^{2}}{k^{2}}$ and $\sigma_{∥}\left(\lambda ,\theta \right)=\frac{{\left|S_{2}\left(\lambda ,\theta \right)\right|}^{2}}{k^{2}}$ are the particle differential scattering cross‐sections for orthogonal and parallel polarizations, respectively. We use the ratio of $\sigma_{⊥}$/$\sigma_{∥}$ to eliminate the transmittance of the sample *T*(*λ*), the source intensity *I*_0_(*λ*), and the instrumental variations that are wavelength and angle‐dependent (represented by the *g*(*λ*, *θ*) function) in the Equation (S2) and (S3), Supporting Information, which allows the data to be directly analyzed without the need for normalization.^[^ ^26^ ^]^

A supercontinuum laser (SuperK EXB‐6 with SuperKSplit filter from NKT Photonics) emitting from 440 to 900 nm was used as the light source. The incident beam is linearly polarized by a Glan‐Taylor polarizer (Thorlabs GL5‐A) and the polarization angle *φ* is controlled by the motorized rotation of an achromatic half‐wave plate (Thorlabs SAHPWP05M‐700).

The sample consisted of a colloidal suspension of Si@SiO_2_ core–shell particles in chloroform placed in a cuvette of fused silica. The dilution factor was important and selected to avoid multiple scattering events. The scattered light was collected at *θ* = 45°, 90°, and 135° with respect to the direction of the incident light, using a Minispectrometer (Hamamatsu C10083CA) fed with a collimated optical fiber. A second Glan‐Taylor polarizer (Thorlabs GL10‐A), placed between the sample and the detector, analyzed the scattered light with a polarization parallel (axial) or perpendicular (transverse) to the scattering plane. Scattering spectra are collected over a 360° rotation of the *φ* angle at intervals of 10°. The scattering intensities measured along the transverse and axial directions are the following:

(3) $$I_{\text{sca,}⊥}I\;\left(\lambda ,\theta ,\varphi \right)=\;I_{0}(\lambda )\;\cdot \left(N_{\text{sca}}/k^{2}\right)\cdot |S_{1}(\theta ){|}^{2}\cdot \delta \Omega \cdot \;T(\lambda )\cdot \;g(\lambda ,\theta )\cdot {\text{sin}}^{2}\varphi =\tilde{I_{⊥}}\left(\lambda ,\theta \right)\cdot {\text{sin}}^{2}\varphi$$

(4) $$I_{\text{sca,}\|}\;\left(\lambda ,\theta ,\varphi \right)=I_{0}(\lambda )\cdot \left(N_{\text{sca}}/k^{2}\right)\cdot {\left|S_{2}\left(\theta \right)\right|}^{2}\cdot \;\delta \Omega \cdot T\left(\lambda \right)\cdot g(\lambda ,\theta )\cdot {\text{cos}}^{2}\varphi =\tilde{I_{∥}}\left(\lambda ,\theta \right)\cdot {\text{cos}}^{2}\varphi$$

where *I*_0_(*λ*) is the spectral irradiance of the incident beam, *N*_scat_ represents the number of particles in the scattering volume, and *k* is the wavenumber (2*π*/*λ*). The solid angle δΩ represents the angular detection window, *T*(*λ*) is the transmittance of the suspension, and *g*(*λ*, *θ*) is an unknown function of the instrument, dependent on the spectral power of the source, the sensitivity of the detector and the transmittance and reflectance of all the optical elements.

The experimental axial and transverse scattering have been fitted for each wavelength with functions of the type *B*_∥_ + $\tilde{I_{∥}}\left(\lambda ,\theta \right)$sin^2^ *φ* and *B*_⊥_ + $\tilde{I_{⊥}}\left(\lambda ,\theta \right)$ cos^2^ *φ*, respectively. The background signals *B*_∥_ and *B*_⊥_ measure a residual anisotropy of the scatterers,^[^ ^27^ ^]^ which we find below 10% in most of our samples.

##### Maxwell–Garnett Model

The Maxwell–Garnett model^[^ ^28^ ^]^ (see Equation (1) in the main text) was used to build the effective permeability of the Si core and the shell. The complex effective refractive index, *ñ* = *n* + *iκ*, is the square root of the effective permittivity. We fabricated an effective permittivity by considering the particles to be composed of a mixture of crystalline Si, amorphous Si, and low‐index inclusions. The index dispersion for amorphous and crystalline Si were obtained from the data collected by Pierce and Spicer^[^ ^28^ ^]^ and Aspnes and Studna,^[^ ^29^ ^]^ respectively, available on an online database.^[^ ^30^ ^]^ To simulate the presence of low‐index impurities, included within the particles, we used the refractive index of water.^[^ ^31^ ^]^ This model allows the volume fraction, *f*_m_, of crystalline Si, amorphous Si, and low index impurities to be adjusted to obtain the best agreement between simulations and experiments,^[^ ^32^ ^]^ while respecting the Kramers–Kronig conditions.

##### Ellipsometric Spectroscopy

Ellipsometric spectra were obtained using a J.A. Woollam M–2000F variable angle spectroscopic ellipsometer with a 240–1700 nm spectral range and 1–1.6 nm resolution was used. The light source was a 75 W Xe lamp producing a reasonably smooth UV‐vis‐IR continuum spectrum. Spectroscopic ellipsometry measurements are conducted at incident angles of 50°, 55°, 60°, 65°, and 70° in the reflectance mode. The M–2000F uses a rotating compensator that ensures high accuracy over the complete measurement range. The measured ellipsometric parameters were extracted from fits of the spectra using WVASE32 ellipsometric fitting software. This software allows the modeling of the layer made of dielectric particles as a Fresnel effective medium layer with non‐trivial permittivity and permeability (a so‐called Meta6 layer). It then adjusts all six constants of this layer (per given wavelength), corresponding to the complex permittivity, permeability, and chirality to achieve the best fit to the measured data.

## Conflict of Interest

The authors declare no conflict of interest.

## Author Contributions

**Megan A. Parker**: investigation (equal); writing—original draft (supporting). **Raul Barbosa**: formal analysis (supporting); investigation (supporting). **Cynthia Cibaka‐Ndaya**: formal analysis (supporting). **Alexander Castro‐Grijalba**: investigation (supporting). **Maria Letizia De Marco**: methodology (equal). **Brian A. Korgel**: resources (supporting); supervision (supporting). **David Montero**: investigation (supporting). **Sabrina Lacomme**: investigation (supporting). **Antoine Azéma**: investigation (supporting). **Vasyl G. Kravets**: investigation (supporting). **Alexander N. Grigorenko**: formal analysis (supporting); resources (supporting); visualization (supporting); writing—original draft (supporting); writing—review & editing (supporting). **Virginie Ponsinet**: investigation (supporting); resources (supporting); writing—original draft (supporting); writing—review & editing (supporting). **Philippe Barois**: conceptualization (supporting); formal analysis (equal); investigation (supporting); resources (supporting); writing—review & editing (supporting). **Lucien Roach**: formal analysis (equal); investigation (supporting); methodology (equal); software (lead); visualization (equal); writing—original draft (supporting). **Glenna L. Drisko**: conceptualization (lead); formal analysis (supporting); funding acquisition (lead); methodology (supporting); project administration (lead); resources (lead); supervision (lead); visualization (supporting); writing—original draft (lead); writing—review & editing (equal).

## Supporting information

Supplementary Material

## Data Availability

The data that support the findings of this study are openly available in [10.5281/zenodo.15100715] at [https://doi.org/10.5281/zenodo.15100715], reference number [15100715].
