# Supplementary material for: Self‐Assembled Silicon@Silica Metasurfaces with High‐Quality Resonances in the Infrared
Source: Small Sci. 2025 May 15;5(7):2500119. doi: 10.1002/smsc.202500119 (PMC12257874; doi:10.1002/smsc.202500119)
Supplement: Supplementary file 1 — Supplementary Material [file SMSC-5-2500119-s001.pdf]

Supporting Information

Self-Assembled Silicon@Silica Metasurfaces with High-Quality Resonances in the Infrared

Megan A. Parker, Raul Barbosa, Cynthia Cibaka-Ndaya, Alexander Castro-Grijalba, Maria Letizia De Marco, Brian A. Korgel, David Montero, Sabrina Lacomme, Antoine Azéma, Vasyl G. Kravets, Alexander N. Grigorenko, Virginie Ponsinet, Philippe Barois, Lucien Roach, Glenna L. Drisko**

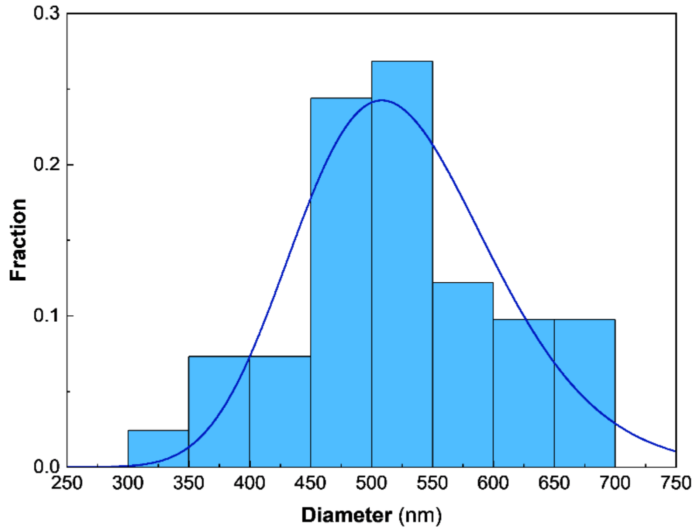

Batch	Size (nm)	Batch	Size (nm)	Batch	Size (nm)	Batch	Size (nm)	Batch	Size (nm)
106B	667±56	106A	587±41	107F	536±31	108G	489±45	73B	451±25
105F	659±56	107B	585±51	109C	536±35	77G	486	82B	433±49
104G	652±42	105G	559±40	99E	527±48	108C	483±41	82G	428±50
102G	629±43	107A	559±35	108B	526±40	108E	478±43	77E	423±62
107D	623±44	107G	546±34	101B	521±41	107C	476±26	109B	398±46
107E	614±44	104E	540±36	106C	521±32	79B	474±58	108F	366±44
104B	608±50	102B	539±37	102F	518±33	109A	459±31	75E	363±24
103B	593±40	103G	539±45	103F	498±35	106D	453±32	81G	328±44

Figure S1. Distribution of silicon particle diameters across 40 different batches prepared under the same conditions, i.e. a batch reactor loaded with *n*-hexane (5.6 mL), cyclohexasilane (5.5 µL, 30 µmol), and bis(*N*,*N*'–diisopropylbutyl)dichlorosilane (28 µL, 10 µmol) placed on a heating block at 460 °C for 10 min. Table of the batches of prepared particles, where at least 100 particles were counted in SEM images taken of the batch. Orange cells represent the batches that were combined and used for the 2D assembly.

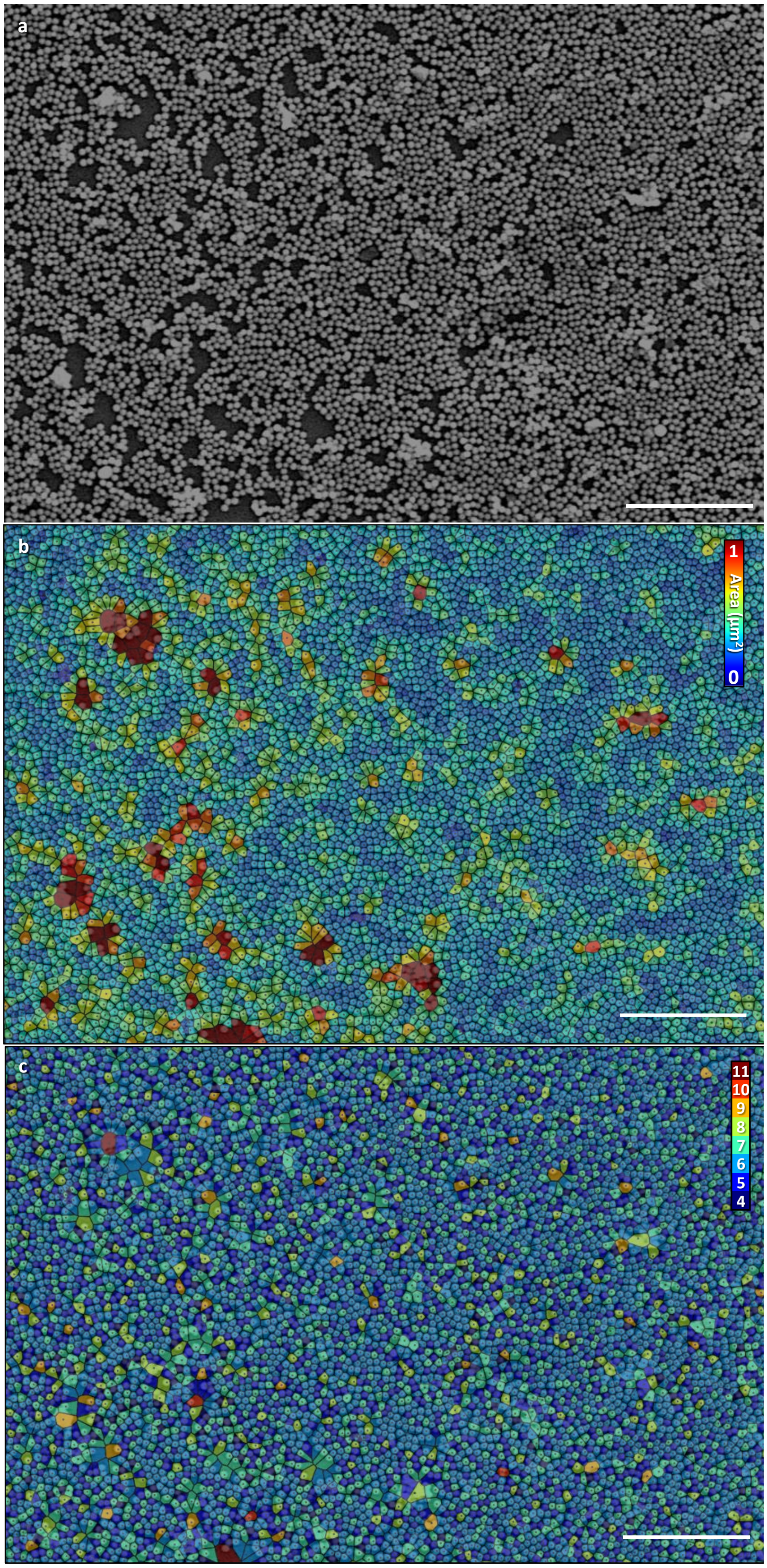

Figure S2. (a) Segmented (binarized) SEM image of a monolayer prepared from a suspension of the core-shell particles in a 2:1 ethanol:butanol mixture at a particle concentration of 2 % (v/v). The scale bar represents 10 µm. (b) The area of the Voronoi cell. (c) The number of nearest neighbors.

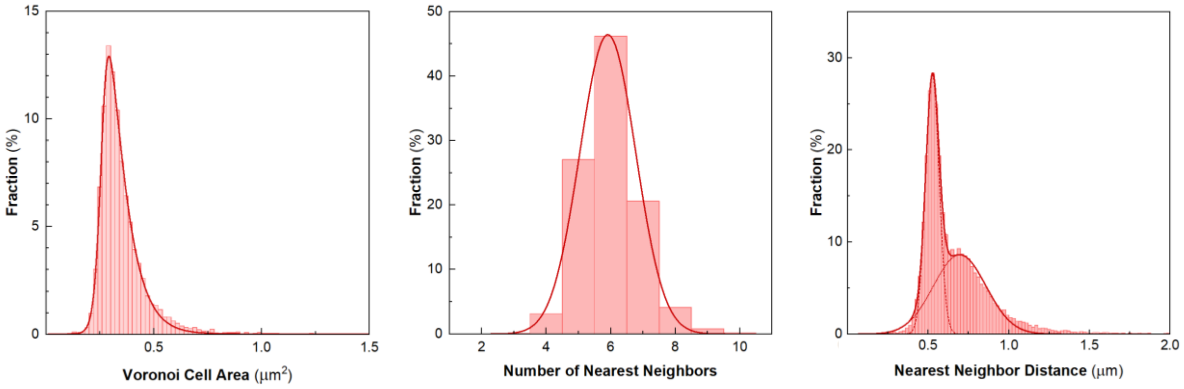

Figure S3. Histogram of (a) Voronoi cell areas, (b) number of nearest neighbors and (c) nearest neighbor distances (Delaunay lengths).

Table S1. Parameters used to simulate representative particles suspended in a solvent having a refractive index of 1.43.

Diameter	Radius		Matrix	Inclusions Si
544 nm	Core	494 nm	c-Si	a-Si 25 %
				H_2O 10 %
	Shell	25 nm	H_2O	c-Si 1%
				a-Si 1%

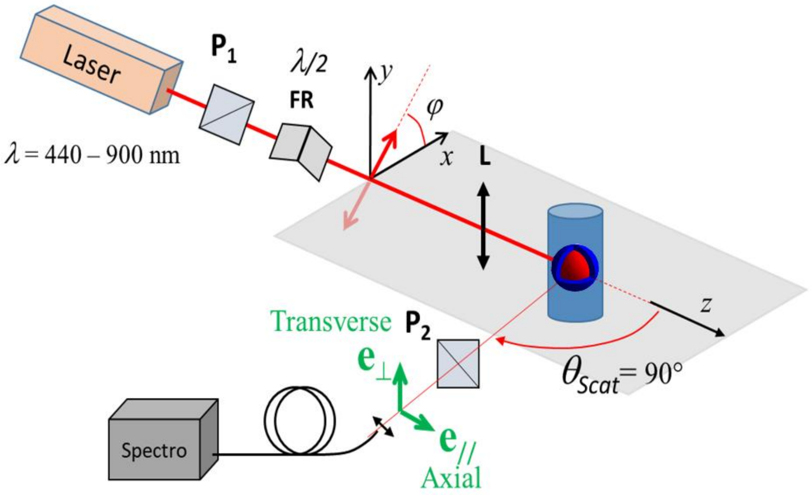

Figure S4. Schematic representation of the static light scattering set-up.

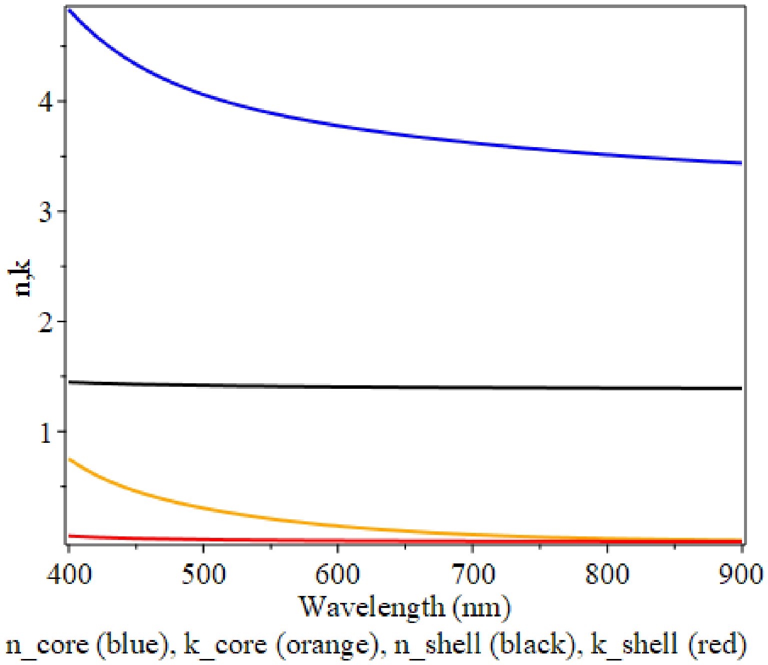

Figure S5. Refractive index and absorption coefficients obtained from fitting the SLS data.

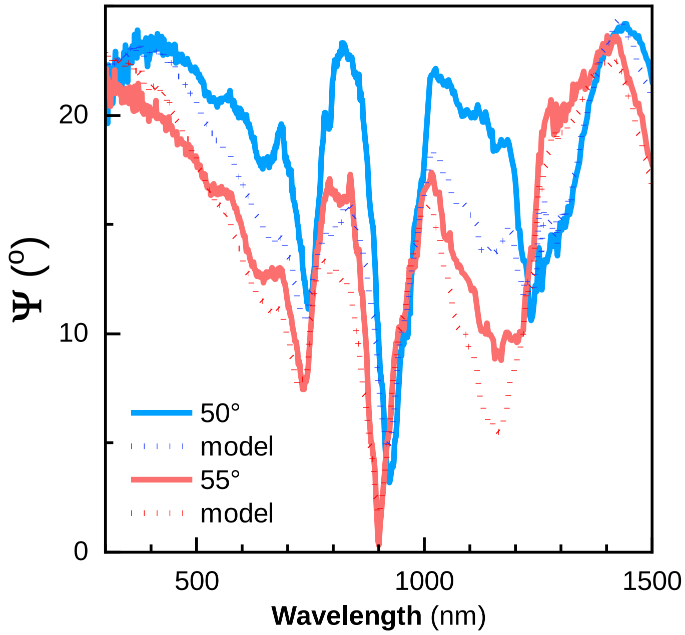

Figure S6. Fresnel fitting of the variable angle ellipsometry spectra using the *Meta6* model with 540 nm diameter particles and a quartz Cauchy substrate of 1 mm at 50° and 55°. Experimental data plotted (solid lines) *vs.* modeled spectra (dotted lines).
